# Curcumin as a potential therapeutic agent for Parkinson’s disease: a systematic review

**DOI:** 10.3389/fphar.2025.1593191

**Published:** 2025-04-22

**Authors:** Yu-Hsien Chang

**Affiliations:** School of Medicine, China Medical University, Taichung, Taiwan

**Keywords:** curcumin, Parkinson’s disease, parkinsonism, neuroprotection, complementary therapy

## Abstract

**Importance:**

Parkinson’s disease (PD) is a growing global health concern with the number of affected individuals projected to double by 2040. Current treatments primarily address motor symptoms but do not prevent disease progression and often have significant side effects.

**Objective:**

To evaluate the clinical efficacy and safety of curcumin as an adjunctive treatment for PD, with a focus on its impact on motor and non-motor symptoms, quality of life, and neuroprotective mechanisms, especially regarding α-synuclein aggregation.

**Evidence Review:**

A systematic search was conducted in Web of Science, Embase, PubMed, CINAHL, and Cochrane Library from February to March 2025, using specific search terms, and following the PRISMA 2020 guidelines. The search strategy used the terms (“Parkinson’s disease” OR “Parkinson Disease” OR “Parkinsonism”) AND (“Curcumin” OR “Turmeric” OR “Diferuloylmethane” OR “Curcuminoids”), limiting results to English-language publications. The Cochrane Risk of Bias 2 Tool was used for assessing the risk of bias in RCTs, and the Newcastle-Ottawa scale was used for the cohort study.

**Findings:**

The review included two randomized controlled trials and one cohort study, comprising a total of 125 PD participants. The studies suggest that curcumin may offer modest benefits as an adjunct therapy in PD when administered in formulations designed to enhance its bioavailability. Long-term curcumin supplementation was associated with a slower deterioration of motor function and a tendency to reduce the deposition of phosphorylated α‐synuclein in skin nerves. A nanomicelle formulation of curcumin significantly improved sleep quality and overall quality of life in PD patients over a three-month period, while no significant effect was observed on fatigue severity.

**Conclusion and Relevance:**

Curcumin, particularly in formulations that enhance its bioavailability, may be a beneficial add-on treatment for PD, potentially improving non-motor symptoms and slowing the advancement of motor dysfunction. However, current clinical practice guidelines do not recommend curcumin due to the limited and preliminary nature of the evidence. Additional validation through larger trials with standardized methodologies is necessary to confirm these findings.

**Systematic Review Registration:**

https://www.crd.york.ac.uk/PROSPERO/view/CRD420251000404.

## 1 Introduction

Parkinson’s disease (PD) is a progressive neurodegenerative disorder marked by the loss of dopaminergic neurons in the substantia nigra pars compacta (Surmeier, 2018), resulting in reduced dopamine production (Damier et al., 1999) and impaired basal ganglia function necessary for coordinated voluntary movement (Simonyan, 2019; Balestrino and Schapira, 2019). Early degeneration of the locus coeruleus further diminishes noradrenergic neurons and norepinephrine levels (Gesi et al., 2000), which is linked to non-motor symptoms such as depression, anxiety, and cognitive decline (Ferreira and Guerra, 2015). At the cellular level, PD is characterized by abnormal accumulation of alpha-synuclein protein into insoluble Lewy bodies (Mitchell et al., 2015) that propagate in a prion-like fashion (Lee and Trojanowski, 2006), leading to synaptic pathology (Kramer and Schulz-Schaeffer, 2007); this process is compounded by oxidative stress, mitochondrial dysfunction, and neuroinflammation (Dias et al., 2013). Genetic factors, including mutations in LRRK2, PARK7, SNCA, and GBA (Pang et al., 2019), as well as environmental toxins like pesticides and metals, also contribute to disease risk (Fleming, 2017). Clinically, PD typically presents with motor symptoms, including resting tremor, bradykinesia, rigidity, dystonia, and postural instability (Alves et al., 2005), often accompanied by non-motor features such as cognitive impairment, sleep disturbances, and autonomic dysfunction (Poewe, 2008; Sklerov et al., 2022). With the number of individuals affected more than doubling between 1990 and 2016, reaching approximately 6.1 million worldwide, PD’s prevalence is projected to double again by 2040, driven by aging populations and longer disease duration (GBD 2016 Parkinson's Disease Collaborators, 2016). PD is a significant and growing global health challenge, and the escalating burden underscores the urgent need for effective therapeutic strategies to manage PD.

Conventional pharmacological treatments, notably levodopa and dopamine agonists, effectively manage motor symptoms but do not halt disease progression and are associated with long-term complications like dyskinesia (Pilleri and Antonini, 2015), prompting the use of advanced interventions such as deep brain stimulation (DBS) and MR-guided focused ultrasound (MRgFUS) in refractory cases (Clarke et al., 2009; Gaspari et al., 2006), as well as non-pharmacological approaches including physical, occupational, and speech therapy, along with nutritional management (Sharpe et al., 2020). However, PD remains progressive, with most patients developing motor fluctuations, postural instability, cognitive decline, depression, and autonomic dysfunction, as well as experiencing increased mortality within 10 years of diagnosis (Williams-Gray et al., 2013), a pooled mortality ratio of 1.5 (Macleod et al., 2014), and a median survival ranging from 6 to 22 years (Marttila et al., 1991). Amid these challenges, there is growing interest in complementary therapies that offer neuroprotective benefits.

Curcumin, a natural polyphenol derived from turmeric, exerts potent antioxidant effects by scavenging reactive oxygen species and upregulating endogenous antioxidant defenses, such as glutathione, thereby mitigating mitigate oxidative stress—a key contributor to neuronal death (Abrahams et al., 2019). Additionally, its anti-inflammatory properties, mediated through the inhibition of pro-inflammatory cytokines and suppression of microglial activation, further protect neurons from inflammatory damage. Moreover, curcumin has been shown to interfere with the aggregation of alpha‐synuclein, thereby potentially reducing the formation of Lewy bodies that are characteristic of PD (Menon and Sudheer, 2007). Preclinical studies suggest that curcumin can prevent neuronal loss in the substantia nigra through scavenging reactive species, upregulating glutathione, and inhibiting α-synuclein aggregation (Sharma and Nehru, 2017; Cui et al., 2016). Despite its inherently low bioavailability, recent advances in formulation technology, such as phospholipid-based, microemulsified, and nanomicelle preparations, have improved its systemic absorption, enhancing its therapeutic potential (Liu et al., 2016). Together, these neuroprotective mechanisms position curcumin as a candidate for alleviating PD symptoms.

This systematic review evaluates the clinical efficacy and safety of curcumin as an adjunctive treatment for PD, with a focus on its impact on motor and non-motor symptoms. The review extracts outcomes of curcumin treatments reported in two randomized controlled trials with a 3-month and a 9-month follow-up and one cohort study with a one-year follow-up.

## 2 Methods

The systematic review protocol was submitted and registered with PROSPERO on 27 February 2025 (registration ID: CRD420251000404). This systematic review followed the PRISMA 2020 guidelines to systematically search for articles in Web of Science, Embase, PubMed, CINAHL, and Cochrane Library from February to March 2025. The search strategy used the terms (“Parkinson’s disease” OR “Parkinson Disease” OR “Parkinsonism”) AND (“Curcumin” OR “Turmeric” OR “Diferuloylmethane” OR “Curcuminoids”), limiting results to English publications. The search for articles did not have any filters for the date of publication.

Inclusion criteria: 1) Studies that include patients diagnosed with PD using recognized diagnostic criteria. 2) Studies that include PD patients on stable doses of dopaminergic medications. 3) Studies that evaluate the effect of curcumin supplementation on PD patients compared to the placebo. 4) Studies that measure motor or non-motor symptoms of PD using recognized measurement tools. Exclusion criteria: 1) Studies that include patients with PD due to atypical parkinsonism or secondary causes. 2) Studies that include patients with severe systemic or psychological disease. 3) Studies with insufficient statistical information for data synthesis.

Cochrane Risk of Bias 2 Tool is used for assessment of the risk of bias of RCTs, while Newcastle-Ottawa scale is used for cohort study. Data was extracted into a pre-defined Microsoft Excel (V2201, Microsoft, Washington, United States) template under the categories of study details. Two reviewers (Yu-Hsien Chang and Yu-Hsuan Chang) independently assessed the reporting quality and extracted data. Any differences in opinion were thoroughly discussed until a consensus was reached.

## 3 Results

### 3.1 Study selection and characteristics

**FIGURE 1:**
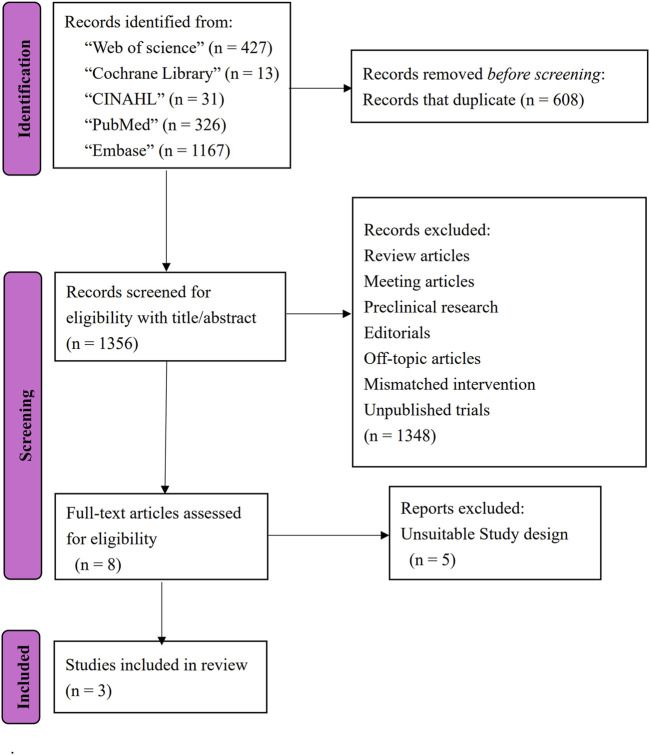
Flow diagram of this systematic review.

A total of 1964 articles were identified through database searches. Yu-Hsien Chang and Yu-Hsuan Chang independently screened the articles, excluding conference abstracts, protocols, reviews, editorials, and preclinical research. Following a full-text assessment of eight articles, two randomized controlled trials (RCT) (Ghodsi et al., 2022; Maghbooli et al., 2019) and one cohort study (Donadio et al., 2022) met the eligibility criteria for inclusion in this review. The flow diagram is shown in Figure 1. These studies comprised a total of 125 PD participants. Table 1 summarizes the characteristics of the included studies, presenting study IDs, patient demographics, interventions, follow-up durations, evaluations, and main outcomes.

**TABLE 1:** Basic information of included studies.

Study ID^a^	Study type	Intervention	Completed participants number	Age, mean (SD), year	Disease duration, mean (SD), year	Levodopa dosage, mean (SD), mg/d	Follow-up duration, month	Evaluation	Outcomes
Maghbooli 2019	Double-Blind, Randomized, Placebo-Controlled Trial	Curcumin Nanomicelle, 160 mg/d	25	67.80 (10.14)	4.36 (2.37)	495.65 (286.79)	3	PSQI^b^ FSS^c^ PDQ-39^d^	● Nanomicelle curcumin significantly increased sleep quality and quality of life compared to placebo (P values = 0.0001 and 0.0002, respectively) in PD patients .● The supplement did not have a significant effect on the fatigue severity of patients compared to placebo
		Placebo	25	63.08 (10.47)	4.68 (3.11)	526.25 (249.27)			
Ghodsi 2022	Pilot, randomized, triple-blind, placebo-controlled trial	Curcumin Nanomicelle, 80 mg/d	23	58.4 (9.4)		562	9	MDS-UPDRS^e^ PDQ-39	● The trial did not show a significant effect of curcumin on the quality of life and clinical symptoms of PD patients ● There was no significant difference between the curcumin and placebo groups in mean MDS-UPDRS and PDQ-39 scores at any time ● MDS-UPDRS part III showed a significant difference in its overall trend between the study groups (P = 0.04), but *post hoc* analysis failed to identify this difference at study time points ● The most common side effects of curcumin were nausea and vomiting (P = 0.25) and gastroesophageal reflux (P = 0.42)
		Placebo	19	58.0 (12.9)		625			
Donadio 2022	Prospective cohort study	Curcumin as curcumin phospholipids, 2 g/d	19	72 (Damier et al.)	5 (Surmeier, 2018)	270 (Sharpe et al., 2020)	12	MDS-UPDRS H&Y^f^ NMSS^g^ COMPASS-31^h^ Skin biopsy	● Supplemented patients showed a decrease in COMPASS-31 and NMSS scores, and a slight p-syn load decrease versus untreated patients, who displayed a worsening of these parameters despite increased levodopa doses ● The regression model showed a significant effect of curcumin supplementation in decreasing the worsening of MDS-UPDRS, H&Y, and NMSS compared to untreated patients (p < 0.001)16
		Placebo	14	72 (Damier et al.)	6 (Surmeier, 2018)	180 (Maghbooli et al., 2019)			

^a^ Study ID represents the name of the first author and year of publication.

^b^ Pittsburgh Sleep Quality Index.

^c^ Fatigue Severity Scale.

^d^ Parkinson’s Disease Questionnaire–39.

^e^ Movement Disorder Society-Unified Parkinson’s Disease Rating Scale.

^f^ Hoehn and Yahr Scale.

^g^ Non-Motor Symptoms Scale.

^h^ Composite Autonomic Symptom Score 31.

### 3.2 Assessment of risk of bias

The included RCTs exhibited a high reporting standard with a low risk of overall bias (Table 2), while the cohort study demonstrated high quality (Table 3).

**TABLE 2:** Detailed quality assessment of the included studies using the Cochrane risk of bias 2 tools.

Study ID	Randomization process	Intervention adherence	Missing outcome data	Outcome measurement	Selective reporting	Overall RoB
Maghbooli 2019	Low risk	Low risk	Low risk	Low risk	Low risk	Low risk
Ghodsi 2022	Low risk	Low risk	Some risk	Low risk	Some risk	Low risk

**TABLE 3:** Quality assessment of the included study with Newcastle-Ottawa Scale.

	Selection				Comparability	Outcome			
Study ID	Representativeness of the exposed cohort	Selection of the non exposed cohort	Ascertainment of exposure	Demonstration that outcome of interest was not present at start of study	Comparability of cohorts on the basis of the design or analysis	Assessment of outcome	Was follow-up long enough for outcomes to occur	Adequacy of follow up of cohorts	Total score
Donadio 2022	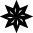		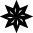	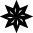	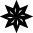 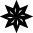	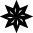	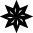	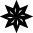	8

### 3.3 Synthesized findings

A pilot randomized, triple‐blind, placebo‐controlled trial evaluated curcumin as an add-on therapy in PD (Ghodsi et al., 2022) found that, despite its favorable tolerability and acceptable safety profile, the supplementation did not produce significant improvements in overall motor function or quality of life as measured by the Movement Disorder Society-sponsored revision of the Unified Parkinson’s Disease Rating Scale (MDS‐UPDRS) and Parkinson’s Disease Questionnaire–39 (PDQ‐39) scores. Although a significant trend was observed in MDS‐UPDRS part III scores over time, *post hoc* comparisons did not confirm significant differences at specific time points. The authors suggested that limitations such as a small sample size and heterogeneity in disease severity might have obscured potential neuroprotective benefits.

In contrast, a subsequent double-blind, randomized, placebo-controlled trial (Maghbooli et al., 2019) focusing on nonmotor outcomes demonstrated that nanomicelle curcumin significantly enhanced sleep quality and overall quality of life in PD patients. Improvements were observed via robust changes in the Pittsburgh Sleep Quality Index (PSQI) and PDQ‐39 scores, whereas fatigue severity remained largely unaffected. These effects appeared to be independent of the disease duration, severity, and levodopa dosage, underscoring the potential advantage of enhanced bioavailability with nanomicelle formulations for targeting nonmotor symptoms.

Extending these findings, the third study (Donadio et al., 2022) explored both clinical outcomes and the pathological substrate of PD by assessing curcumin’s impact on motor and nonmotor symptoms alongside phosphorylated α‐synuclein (p‐syn) accumulation in skin nerve biopsies. Over a 12‐month supplementation period, PD patients receiving curcumin exhibited a slower progression of motor disability and nonmotor impairment, with a notable tendency toward reduced p‐syn deposits compared to untreated patients. These data indicate that curcumin—when formulated for enhanced absorption—can cross the blood-brain barrier and may exert neuroprotective, disease-modifying effects, particularly in patients with shorter disease duration. Collectively, the synthesized findings suggest that while curcumin’s impact on motor symptoms may be limited in heterogeneous PD populations, its improved formulations show promise in alleviating nonmotor symptoms and modifying underlying pathological processes.

## 4 Discussions

The synthesis of the three trials reveals that curcumin may offer modest benefits as an adjunct therapy in PD when administered in formulations designed to enhance its bioavailability. In the study by Donadio et al. (2022), long‐term curcumin supplementation (using a phospholipid formulation) was associated with a slower deterioration of motor function and, importantly, with a tendency to reduce the deposition of phosphorylated α‐synuclein (p‐syn) in skin nerves. In contrast, the pilot randomized, triple‐blind, placebo‐controlled trial by Ghodsi et al. reported a significant difference in the overall trend of MDS‐UPDRS part III scores between the curcumin and placebo groups over 9 months, although total motor scores and quality-of-life measures did not reach statistical significance. Complementing these findings, Maghbooli et al. demonstrated that a nanomicelle formulation of curcumin significantly improved sleep quality and overall quality of life (as measured by PDQ-39) in PD patients over a three-month period, while no significant effect was observed on fatigue severity. Together, these studies suggest that curcumin may have the potential to address nonmotor symptoms and modify certain aspects of disease progression, even if its impact on global motor function remains modest. Table 4 summarizes the effects of curcumin on PD symptoms.

**TABLE 4:** Summary of curcumin treatment effects on Parkinson’s disease symptom.

Supplementation	Ratings of the quality of the evidence	Treatment effect (v.s. Placebo)	Adverse effects	Comments
Curcumin Nanomicelle, 80 mg/d	1	MDS-UPDRS^a^: No significant difference overall, though Part III showed a significant difference in overall trend (P = 0.04), but *post hoc* analysis failed to confirm this PDQ-39^b^: No significant difference	Nausea, vomiting, gastroesophageal reflux	Well-tolerated, but trial was unsuccessful in showing efficacy in quality of life and clinical symptoms of PD patients
Curcumin Nanomicelle, 160 mg/d	1	PSQI^c^: Significant increase (P = 0.0001) PDQ-39: Significant increase (P = 0.0002) FSS^d^: No significant effect (P = 0.42)	Not specified	Improved sleep quality and quality of life in PD patients
Curcumin (2 g/day as curcumin phospholipids)	2	COMPASS-31^e^: Decrease NMSS^f^: Decrease p-syn^g^ load in skin nerves: Slight decrease	Not specified	The data suggests that curcumin can cross the blood-brain barrier and is effective in ameliorating clinical parameters

^a^ Movement Disorder Society-sponsored revision of the Unified Parkinson’s Disease Rating Scale.

^b^ Parkinson’s Disease Questionnaire.

^c^ Pittsburgh Sleep Quality Index.

^d^ Fatigue Severity Scale.

^e^ Non-Motor Symptoms Scale.

^f^ Composite Autonomic Symptom Score.

^g^ phosphorylated α-synuclein.

A critical comparison of study designs and endpoints helps clarify these outcomes. Donadio et al. (2022) employed a prospective, nonrandomized design in which patients were allocated to either a curcumin-supplemented group or a control group based on their treatment preference. Their comprehensive assessment included motor scales (MDS-UPDRS and Hoehn and Yahr), nonmotor scales (Non-Motor Symptoms Scale, NMSS and Composite Autonomic Symptom Score 31, COMPASS-31), and a novel biomarker approach using skin biopsies to quantify p-syn load. Notably, the curcumin group not only experienced a slower worsening of motor symptoms despite a fixed levodopa dosage but also exhibited a trend toward reduced pathological α-synuclein deposition, particularly among patients with a shorter disease duration. In contrast, the trial by Ghodsi et al. (2022), which utilized a rigorous randomized, triple-blind methodology, focused primarily on motor outcomes (MDS-UPDRS) and quality of life (PDQ-39) over 9 months. Although the overall MDS-UPDRS total scores were not significantly different between groups, the significant trend in part III (motor examination) suggests that curcumin may have a differential effect on specific motor functions. Meanwhile, Maghbooli et al. concentrated on nonmotor outcomes, using well-validated instruments such as the PSQI and PDQ-39 to capture improvements in sleep and quality of life over a shorter treatment period. Their findings of enhanced sleep quality and quality of life provide evidence that curcumin’s benefits may be most pronounced in the realm of nonmotor symptoms.

To mitigate potential biases inherent in prior studies on curcumin and PD, this review applied standardized risk-of-bias assessment tools (Cochrane Risk of Bias 2 for RCTs and the Newcastle-Ottawa Scale for cohort studies), along with dual independent review processes for study selection and data extraction. These procedures ensured rigorous quality control and helped identify methodological strengths and limitations within the included studies. The strengths of these studies extend beyond their clinical outcomes. The use of blinded, placebo-controlled designs in the Ghodsi and Maghbooli trials enhances the internal validity of their findings, despite relatively small sample sizes. Donadio et al.’s incorporation of a biomarker (p-syn load in skin nerves) offers a translational link between peripheral pathology and clinical outcomes, which is a connection that may prove valuable in future studies aimed at understanding disease modification.

Another notable strength lies in the attempt to overcome one of curcumin’s most notorious limitations, its poor oral bioavailability, by employing enhanced formulations. The phospholipid-based preparation used by Donadio et al. and nanomicelle formulations employed in the studies by Ghodsi et al. and Maghbooli et al. have demonstrated improved systemic absorption, which is critical to achieving therapeutic effects. These enhanced delivery systems allow curcumin to reach sufficient concentration in the systemic circulation, increasing the likelihood of central nervous system penetration and observable clinical benefit.

Beyond lipid-based delivery systems, other advanced formulation strategies have emerged to further enhance curcumin’s solubility and therapeutic potential. One promising approach is the development of amorphous solid dispersions (ASDs), which maintain curcumin in a high-energy amorphous state to significantly increase dissolution rate and systemic bioavailability without altering the compound’s partition coefficient, therefore preserving curcumin’s native distribution profile across biological membranes, including the blood-brain barrier (Chuah et al., 2014; Zhang et al., 2018a; Parikh et al., 2018; Wang et al., 2022). Various polymeric carriers such as polyvinylpyrrolidone (PVP) (Samsoen et al., 2024), Eudragit (Wang et al., 2022), and hydroxypropyl methylcellulose (HPMC) (Ishtiaq et al., 2022) have been utilized to stabilize curcumin within ASDs through strong molecular interactions, enhancing both solubility and chemical stability. Surfactants like TPGS and co-formers including amino acids (e.g., tryptophan) can further aid in maintaining the amorphous state and improving dissolution (Xi et al., 2023; Garbiec et al., 2025). In addition, advanced techniques like hot-melt extrusion (Wdowiak et al., 2024) and supercritical fluid technology (Garbiec et al., 2025) have been used to optimize the dispersion process and improve permeability across biological barriers. In contrast, traditional powder forms of curcumin or natural turmeric preparations offer minimal clinical benefit and exhibit extremely low absorption, due to their extremely low aqueous solubility, chemical instability, and rapid metabolism (Sabet et al., 2021). Recognizing these formulation distinctions is crucial not only for interpreting the efficacy of curcumin in clinical trials, but also for guiding future pharmaceutical development and regulatory consideration. Although lipid-based and ASD formulations significantly enhance curcumin’s bioavailability, their accessibility and cost-effectiveness in clinical practice remain uncertain. Advanced delivery systems—such as polysorbate 80-modified nanoparticles (Zhang et al., 2018b) and curcumin-levodopa co-loaded biodegradable carriers (Mogharbel et al., 2022)—have demonstrated promising pharmacokinetic and synergistic effects in preclinical models. However, these technologies may be associated with increased production costs and regulatory hurdles, limiting their immediate clinical translation. Ensuring the feasibility of routine clinical use will require further investigation into scalable manufacturing, approval pathways, and reimbursement strategies. Future research should assess not only therapeutic efficacy but also the real-world cost-effectiveness of curcumin-based interventions in diverse healthcare settings.

There are several limitations that temper the interpretation of findings within these three studies. First, the sample sizes in all three studies are small, which limits the statistical power to detect treatment effects. This limitation is particularly critical in studies involving heterogeneous patient populations. For instance, the Donadio et al. (2022) study included patients with a wide range of disease durations and severities, potentially obscuring subgroup differences. None of the included studies conducted stratified analyses based on disease stage, duration, or comorbidities, which may mask subgroup-specific responses to curcumin. These methodological gaps substantially restrict the generalizability of the findings and underscore the need for larger, multicenter randomized trials with stratified designs to better assess curcumin’s efficacy across diverse PD populations. Second, while the randomized designs of the Ghodsi and Maghbooli trials strengthen causal inference, the relatively short follow-up periods (9 months and 3 months, respectively) limit our understanding of long-term effects and sustainability of benefits. Long-term safety and sustained therapeutic benefit cannot be inferred from the current data, emphasizing the need for extended follow-up durations in future research. Third, although the biomarker approach in Donadio et al.’s trial is innovative, the clinical relevance of changes in skin p-syn deposition relative to central neurodegenerative processes remains to be fully established.

While curcumin formulations have demonstrated potential benefits, such as improved sleep quality and attenuation of motor deterioration, significant improvements in motor symptoms or fatigue severity were not observed consistently across studies. This inconsistency may stem from several methodological and clinical factors, including small sample sizes, short treatment durations, and variability in curcumin dosage, formulation type, baseline disease severity, and outcome measurement tools. For example, Ghodsi et al. reported no significant improvements in clinical outcomes, which may be attributable to differences in treatment protocols and endpoint selection. Additionally, the lack of stratified analyses based on disease severity or comorbidities may have obscured subgroup-specific responses. These observations highlight the preliminary nature of the current evidence and emphasize the need for harmonized methodologies, standardized endpoints, and longer-term trials to accurately evaluate curcumin’s therapeutic potential in PD. Additionally, as this review included only three eligible clinical studies with varying designs, outcome measures, and treatment durations, meta-analysis was not feasible. Thus, the findings should be interpreted cautiously, and more robust clinical trials are needed to draw definitive conclusions.

Current clinical practice guidelines, such as those from the MDS (Seppi et al., 2019; Fox et al., 2018), Taiwan Movement Disorder Society (TMDS) (Treatment Guideline Subcommittee of Taiwan Movement Disorder Society, 2023), and (National Institute for Health and Care Excellence, 2017) (NICE) (Wang et al., 2022) predominantly focus on dopaminergic therapies to manage motor symptoms in PD. These guidelines do not currently recommend complementary treatments like curcumin due to the limited and preliminary nature of the evidence (Comparative Evaluation of PD Clinical Guidelines is shown in Table 5). However, the findings from these three trials indicate that curcumin, particularly when delivered in advanced formulations that overcome bioavailability challenges, may have a role in improving nonmotor symptoms and potentially moderating disease progression. Although the effects on motor symptoms are less clear, the apparent stabilization of motor decline in some studies, despite a constant levodopa dosage, suggests that curcumin might exert a neuroprotective effect. At present, the evidence is not sufficiently robust to warrant changes in clinical guidelines, but it does highlight an area in need of further investigation.

**TABLE 5:** Comparative evaluation of Parkinson’s disease clinical guidelines (MDS, NICE, TMDS).

	MDS	NICE	TMDS
Publishing Organization	International Parkinson and Movement Disorder Society (MDS)	National Institute for Health and Care Excellence (NICE), United Kingdom	Taiwan Movement Disorder Society (TMDS)
Last Updated	2018 (motor), 2019 (non-motor)	2017 (updated 2025)	2023
Establishing transparency	Good	Good	Fair
Management of conflict of interest (COI) in the Guideline Development Group (GDG)	Good	Good	Fair
GDG Composition	Good	Good	Good
Clinical practice guideline–systematic review intersection	Good	Good	Good
Establishing evidence foundations and rating strength for each of the guideline recommendations	Good	Good	Good
Articulation of recommendations	Good	Good	Good
External review	Good	Good	Fair
Updating	Good	Good	Fair
Implementation Issues	Good	Good	Good
Motor Symptoms Treatment Highlights	Dopamine agonist (non-ergot), levodopa Immediate Release, MAO-B inhibitors clinically useful; COMT inhibitors and DBS for motor fluctuations recommended	First-line treatment: levodopa; adjunct therapy with dopamine agonists, COMT inhibitors, or MAO-B inhibitors for motor fluctuations	Early PD: preference for dopamine agonists in younger patients; levodopa preferred in older patients (>70); adjunctive therapies (COMT inhibitors, MAO-B inhibitors) for motor fluctuations
Non-motor Symptoms Treatment Recommendations	MAO-B inhibitors and dopamine agonists recommended for some NMS; limited efficacy of SSRIs and TCAs for depression; rivastigmine useful for dementia and apathy	Cholinesterase inhibitors (rivastigmine) recommended for dementia; cautious use of quetiapine and clozapine for psychosis; modafinil considered for daytime sleepiness	Pramipexole useful for PD-related depression; cholinesterase inhibitors beneficial for cognitive dysfunction and gait; quetiapine possibly useful for psychosis
Recommendations on Neuroprotective Therapies	No established clinically useful neuroprotective treatments identified	No clinically proven neuroprotective therapies: neuroprotective therapies not currently recommended	No current evidence supporting disease-modifying therapies

Unresolved issues remain regarding the optimal dosing regimen and formulation of curcumin. The studies reviewed employed different doses, and it is unclear whether a dose-response relationship exists. Furthermore, the variability in treatment duration raises questions about the necessary treatment length to observe sustained benefits. It is also imperative to clarify whether curcumin’s observed benefits are primarily due to its antioxidant and anti-inflammatory properties or whether additional mechanisms, such as the inhibition of α-synuclein aggregation, play a significant role. The interplay between curcumin and conventional PD medications also warrants careful examination, as potential pharmacokinetic or pharmacodynamic interactions could influence both efficacy and safety.

Future research should prioritize large-scale, multicenter randomized controlled trials using standardized outcome measures and stratified patient populations to validate these preliminary findings and determine which subgroups of PD patients might derive the greatest benefit from curcumin supplementation. Such studies should aim to identify optimal dosing regimens, assess long-term safety, and explore pharmacokinetic interactions with conventional therapies. In addition, mechanistic investigations linking peripheral and central biomarkers will be essential for elucidating curcumin’s potential disease-modifying effects.

This review was conducted using a comprehensive and up-to-date search strategy across five major databases and followed the PRISMA 2020 guidelines to ensure methodological rigor and currency of evidence. To our knowledge, this is the first systematic review to critically examine the clinical efficacy of curcumin in PD patients while incorporating the role of advanced formulation technologies in enhancing bioavailability. It synthesizes clinical outcomes, formulation science, and biomarker evidence, thereby offering a translational perspective that bridges pharmacological development with clinical relevance. In conclusion, curcumin, especially in formulations that boost its bioavailability, may serve as beneficial add-on treatment for PD, potentially improving non-motor symptoms and slowing the advancement of motor dysfunction. The current evidence is preliminary, necessitating additional validation through larger trials with standardized methodologies to confirm these findings.

## Data Availability

The original contributions presented in the study are included in the article/supplementary material, further inquiries can be directed to the corresponding author.
